# Deep learning accelerated discovery of photonic power dividers

**DOI:** 10.1515/nanoph-2022-0715

**Published:** 2023-03-03

**Authors:** Gandhi Alagappan, Ching Eng Png

**Affiliations:** Agency for Science, Technology, and Research (A-STAR), Institute of High-Performance Computing, Fusionopolis, 1 Fusionopolis Way, #16-16 Connexis, Singapore 138632, Singapore

**Keywords:** computational photonics, deep learning, photonics

## Abstract

This article applies deep learning-accelerated inverse design algorithms and discovers a spectrum of photonic power dividers with exceptional performance metrics despite the simplicity in the design geometry. The deep learning models exhibit high precisions on the order of 10^−6^ to 10^−8^ for both TE and TM polarizations of light. These models enable ultrafast search for an empirically describable subspace that simultaneously satisfy compact footprints, ultralow losses, ultrawide bandwidth, and exceptional robustness against fabrication randomness. We demonstrate a spectrum of devices for silicon photonics with programmable power splitting ratios, excess losses as small as 0.14 dB, to the best of our knowledge, the smallest footprints on the scale of sub-*λ* ^2^, and low loss bandwidths covering the whole telecommunication spectrum of O, S, E, C, L and U-bands. The robustness of the devices is statistically checked against the fabrication randomness and are numerically verified using the full three-dimensional finite difference time domain calculation.

## 1 Introduction

A wide deployment of low cost and low power consumption photonic applications are only possible if amalgamation of necessary photonic components takes places in a single miniaturized integrated optical chip. An important component in the implementation of integrated optical chip is the photonic power divider [1–18] which splits the incoming light from the input waveguide to the output waveguides in a specified power ratio. This is a key functionality that enables smooth power distribution [19, 20], signal monitoring [21], and signal feedback [22]. Designing low loss, compact and large bandwidth power dividers are thus can be revolutionary in developing miniaturized on-chip integrated photonic devices. Waveguide power dividers have been used in realizing on-chip optical power taps [23], optical filters [24], and integrated quantum optical circuitries for modern implementations of Hanbury Brown and Twiss (HBT) and Hong–Ou–Mandel (HOM) effects [25–28]. This article applies deep learning-accelerated inverse design algorithms and discovers a spectrum of photonic power dividers with exceptional performance metrics despite the simplicity in the design geometry.

Deep learning techniques have received a great deal of attention in many areas of computational photonics, primarily as a tool for design and discovery [29–37]. In the inverse design of a power divider with a specific power splitting ratio, the optimization algorithm [38] accesses the electromagnetic numerical solver multiple times with a count on the scale ranging to thousands. Given the perspectives of the current machine learning era, such repeated explorations would utilize compute resources inefficiently. Any finite parameter space can be captured, and an effective representation can be formulated. Such representation often assumes deep learning models and have been proven to be a solid replacement for the traditional models. The same representation can be used multiple times for designing power divider with various power splitting ratio. Furthermore, the design acceleration provided by this approach enables one to try many different optimization algorithms to satisfy the design specification. In this article, we introduce a framework that encompasses the deep learning-based forward models and accommodates multiple optimization algorithms.

Power dividers can be implemented using directional couplers (DCs) or multimode interference couplers (MMIs). DCs are inevitably long and wavelength-dependent [1, 2], and traditional rectangular MMIs on the other hand, are susceptible to geometrical constraints such as sharp corners [3, 4]. In recent years, inverse design methods have been applied to the design of compact power dividers. Table 1 summarizes the state-of-art literatures for the design of photonic Y-junction (1:1 power ratio) on the standard silicon-on-insulator platform with 220 nm silicon thickness. The problem of sharp corners in rectangular MMIs can be eliminated by mean of segmenting a symmetric MMI into a set of strips and fixing the length of strip [5] via algorithms such as particle swarm optimizations. Similar design also can be achieved using the adjoint-based method coupled with a level-set geometrical parameterization [6]. These methods offer a compact and low loss power divider; however, it operates only for a single power ratio and notably the many segments design generates fine features that invalidates the device design robustness. Alternatively, circular-arc asymmetric Y-junctions are proposed [9] and the device can be tailored to have an arbitrary power splitting ratio by adjusting the radius of the arc. The radius can be as small as 60 nm for small power splitting ratio. Various designs that use regular and irregular two-dimensional (2D) hole arrays on the traditional MMIs [8, 10–12] were reported. The holes have either circular or square cross sections, and their positions are optimized using inverse algorithms that employs advanced methods such as deep learning, direct binary search, nonlinear fast search, and reinforcement learning. Y-junctions designed using with hole arrays are usually bigger and exhibit slightly higher losses but can be constrained to operate at a much wider bandwidth [10, 12]. While hole arrays are exotics, they contain strenuous fabrication features such as circular holes of varying radii below 50 nm (see Table 1) [10–12]. Thus, they require tedious fabrication efforts to realize and hence exhibit poor fabrication robustness.

**Table 1:** Previous designs of compact equal power photonic Y-junctions on the standard single mode silicon-on-insulator platform with a silicon thickness of 220 nm. The table is compiled using the reported values from Refs. [5–13]. The footprint is defined as the rectangular area that encapsulates the maximum extents of the Y-junction.

Reference	Footprint	Loss	Bandwidth	Structure	Design method
	(λ^2^)	(dB)	(nm)		
2013 Zhang [5]	1.17	∼0.1	80	13 strips of varying width	Particle swarm + 2D FDTD
2013 Keraly [6]	1.17	∼0.1	100	Final structure is similar to 2013 Zhang	Adjoint method
2014 Deng [4]	1.12	∼0.3	60	Rectangular MMI	–
2016 Lu [7]	3.08	∼3.4	60	22 × 22 square pixels of 120 nm edges	Direct binary search algorithm coupled with 3D FDTD
2017 Xu [8]	5.4	∼1	30	30 × 30 square pixels of 120 nm edges	Nonlinear fast search method coupled with 3D FDTD
2019 Lin [9]	1.34	∼0.4	100	Circular arcs, many free parameters	–
2019 Tahersima [10]	2.81	∼0.5	200	20 × 20 hole vectors hole diameter 90 nm	Deep learning (deep neural network with feedforward connections)
2020 Wang [11]	2.81	∼0.3	40	20 × 20 hole vector. Varying hole diameters (60–90 nm)	Digitized adjoint method
2020 Tang [12]	2.11	∼0.5	550	20 × 20 hole vector. Varying hole diameters 42–77 nm	Generative deep learning

As we can see from Table 1, the world smallest silicon power dividers have footprints on the scale of $∼1.1\lambda^{2}$ [4–6] and exhibit sub-0.3 dB losses. These devices display fine [5, 6] and sharp [4] features, and as a result the losses can easily vary due to a random fabrication shift. For the bandwidth, device reported in Ref. [12] has the widest low loss bandwidth of 550 nm. However, it has a footprint of $∼2.1\lambda^{2}$ and contains small circular holes of varying radii which demands strenuous fabrications to realize. Here, we report an asymmetric waveguide taper that has the most simplistic geometry in comparison to all previous demonstrations. The width profile of the proposed geometry is constructed using cubic spline interpolation [39] and has only two parameters controlling the taper widths. The structure has neither fine features nor sharp corners. The asymmetry in the widths’ programs the required power splitting ratio. Applying a deep learning-assisted inverse design discovery algorithm in the design space, we arrived at an empirically describable subspace that to the best of our knowledge, has the smallest footprints (on the order sub-wavelength squared); while also offering programmable power splitting ratio, ultralow loss (smaller than 0.3 dB), ultra-wide bandwidth (covering all telecommunication bands, 1260 nm–1675 nm) and high manufacturing feasibility with extra-ordinary robustness against fabrication fluctuations and randomness. The robustness is verified by mean of a statistical study and the details are presented. For a tolerance level as large as 50 nm in the critical parameter, the standard deviation of the loss distribution of our devices is half of the standard deviation of the earlier state-of-art [5]. The simultaneous satisfaction of the performance metric (low loss, small footprints, and wide bandwidth, high robustness) is made possible mainly by two factors. The first is the geometric smoothness and simplicity, and the second is the deep learning-assisted optimization algorithm which conducts an ultrafast search on the entire parameter space. Without deep learning and using traditional approach, the full-fledged optimization presented in this article would have taken days, and thus would have been obstructive for extracting meaningful empirical details. To give a taste of time durations of inverse design using optimization algorithms (i.e., fast search algorithms, and evolutionary algorithms) without deep learning a few examples are cited here. For the case of photonic crystal cavity design using genetic algorithm a week-long optimization is reported in Ref. [40]. Reference [8] reports an average duration 120 h to design a silicon power divider using the nonlinear fast search algorithm. For silicon power divider design with a finite 2D hole array, the conventional direct binary search algorithm consumes a duration on the range of 36 h [11]. In this article, to the direct contrast, we report an unprecedented design acceleration of silicon power dividers with time duration on the scale of seconds. Design acceleration is extremely critical as it allows full exploration of the solution space and enables switching between different optimization algorithms to identify local and global design solutions.

The rest of the article is organized as follows. In Section 2, we present the details of the photonic power divider geometry, and details of the deep learning assisted inverse design algorithm. Section 3 presents the inverse designs of power dividers with arbitrary power splitting ratio. In Section 4, using the equal power Y-junction as an example, we describe the simultaneous satisfaction of performance metrics that consists of footprint, loss and bandwidth. Section 5 describe the robustness of our devices against the fabrication randomness, and Section 6 concludes the article.

## 2 The algorithm

In photonics, there are two general classes of deep learning models used in the device design and discovery [30, 31]. These models use either discriminative neural networks [41–45] or generative neural networks [46–48]. Discriminative neural network is capable of mapping complex relationship between the input and output quantities. Generative neural networks as the name suggest can generate new data points by learning the initial dataset. Generative models are good starting point for inverse design of topologically complex photonic structures with limited data points. Inverse design using discriminative neural networks, on the other hand, can be broadly categorized into three different classes [30]. The first class employs a direct method in which the input and output of the neural network are setup as the response of the device and the geometry of the device, respectively. The technique although sounds straightforward, it suffers from the problem of one-to-many relationship between the response and the geometry. This method has been applied in the design of plasmonic metasurface by limiting the design space to a subspace that possesses a unique, one-to-one relationship [41]. The second class of method employs neural network with input and output being the photonic geometry and the response, respectively [42, 43]. In this method, an educated geometry is evaluated using the trained neural network and the loss function is calculated. The loss is minimized using backpropagation algorithms and by modifying the input geometry. This method delivers a local design solution in the vicinity of the educated guess and therefore unable to arrive at global solutions. The third method is a hybrid approach in which both deep learning and traditional optimization algorithms [38] are employed. Optimization algorithm such as genetic algorithm targets a larger solution space and thus able to provide global solutions. In the hybrid approach, trained neural networks work as fast forward models replacing the numerical electromagnetic solver. This method previously has been applied the design optimization of microwave filters [44] and multilayer thin films [36]. In this article, we adapted the third method to the design an integrated on-chip waveguide power divider. The approach is very advantageous for design of power dividers as setting up the forward model is just one time cost. Multiple robust power dividers with varying power splitting ratio are then can be designed using multiple optimization algorithms that explores the full design space in a rapid manner.

**Figure 1:**
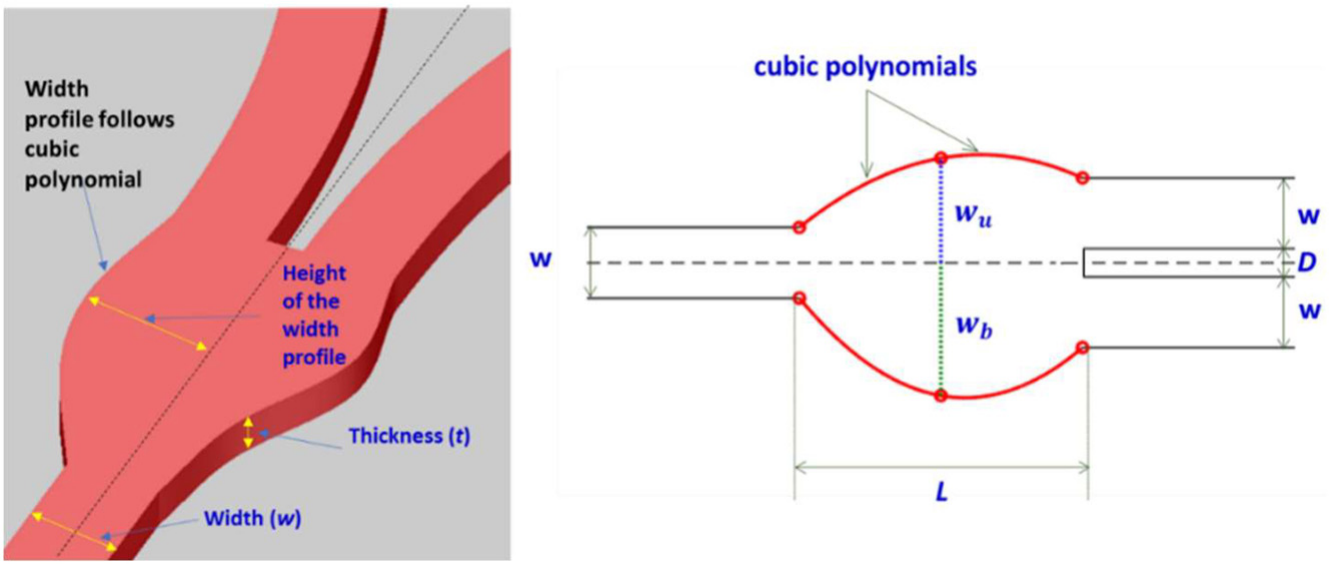
Schematic of the photonic power divider. The core material is silicon, and the cladding material is silica. (Left panel): three-dimensional illustration. (Right panel): planar layout.

Figure 1 portrays the schematic of a photonic power divider for a standard single mode silicon waveguide with width and thickness of *w* = 500 nm and *h* = 220 nm, respectively. The two output waveguides are separated with a distance *D* = 200 nm at the beginning. Thereafter, the two waveguides are extended far from each other by means of s-bends (not shown in the schematic) to avoid evanescent coupling. The input and output waveguides are connected by a divider region of length *L*. For an easier and reproducible fabrication, the divider has a smooth continuous upper and lower tapered width profiles constructed by a cubic spline interpolation method. The heights of upper and bottom width profiles are denoted with design parameters *w*_u_, and *w*_b_, respectively.

**Figure 2:**
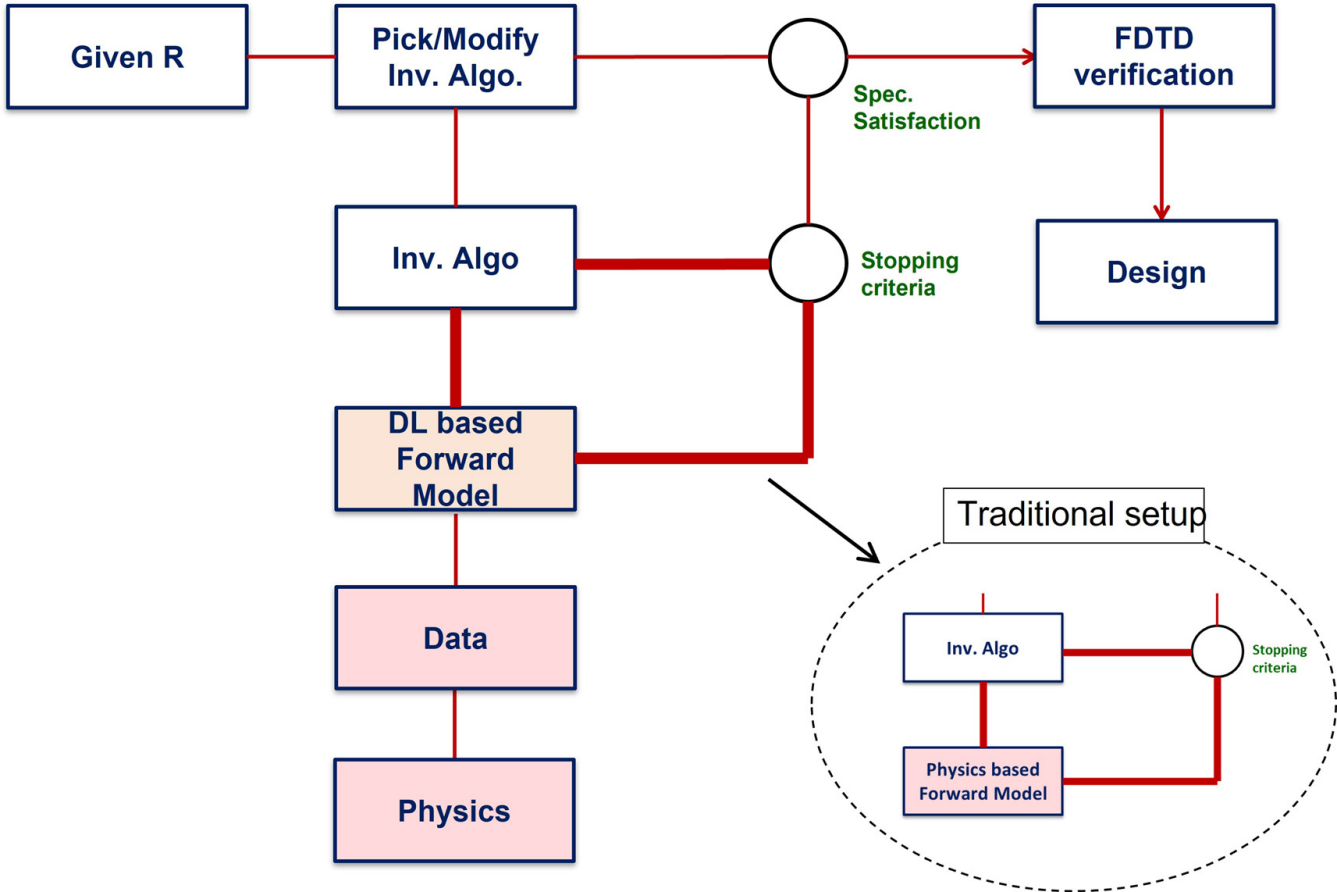
The inverse design framework. This framework blends physics, deep learning (DL), and inverse design under a single umbrella. The computationally expensive physics simulations are moved out from the critical loop (bold red line) to generate data and perform one-time training of the DL models. The trained DL models then bring efficiencies to massively speed up the iterative design process.

Figure 2 illustrates the general framework for the design of photonic power divider with the targeted transmission ratios of the lower and upper waveguides being *R* and 1 − *R*, respectively. The framework takes *R* as input and begins with a selection of inverse design algorithm. The selected inverse algorithm will seek the appropriate geometry [*w*_u_, *w*_b_, and *L*] for the given *R*. In this search, the inverse algorithm (i.e., optimization algorithms [38]) will access the forward model multiple times (hundreds to thousands) in a loop (critical loop – shown in bold line in Figure 2) until a stopping criterion is met. The transmissions of the resulting geometry will be checked against the design specification, and if needed another inverse algorithm can be selected, or the present algorithm can be modified to adjust the stopping criterion of the critical loop. The main bottleneck in the practical implementation of this framework is the amount of time spent in critical loop where the inverse algorithm accesses the forward model multiple times.

Forward model of a power divider calculates the transmissions of the two output waveguides for a given set of geometrical parameters. Traditionally this is done by evaluating Maxwell equations by means of numerical techniques such as finite element [49] and finite difference time domain (FDTD) [50] methods. These techniques discretize the dielectric profile of the divider and implement numerical differentiations to evaluate the resulting equations. A typical FDTD based numerical evaluation can easily take 3–5 min of computational time for each forward calculation [51]. This computational time may look short, but for a count on the scale of hundreds and thousands, the time to exit the critical loop can add up to hours or even days cumulatively [8, 11, 40], and hence it is clearly a bottleneck in the full exploration of the parameter space. Here, instead of the physics-based numerical model, we used a deep learning based forward model that can perform the forward calculation in a split of a second. The computationally expensive physics-based numerical calculations are taken out of the critical loop to generate data and perform one-time training of the deep learning model (see Figure 2). The speed comparison between the deep learning and traditional physics-based numerical forward models will be discussed in detail in Section 3.

**Figure 3:**
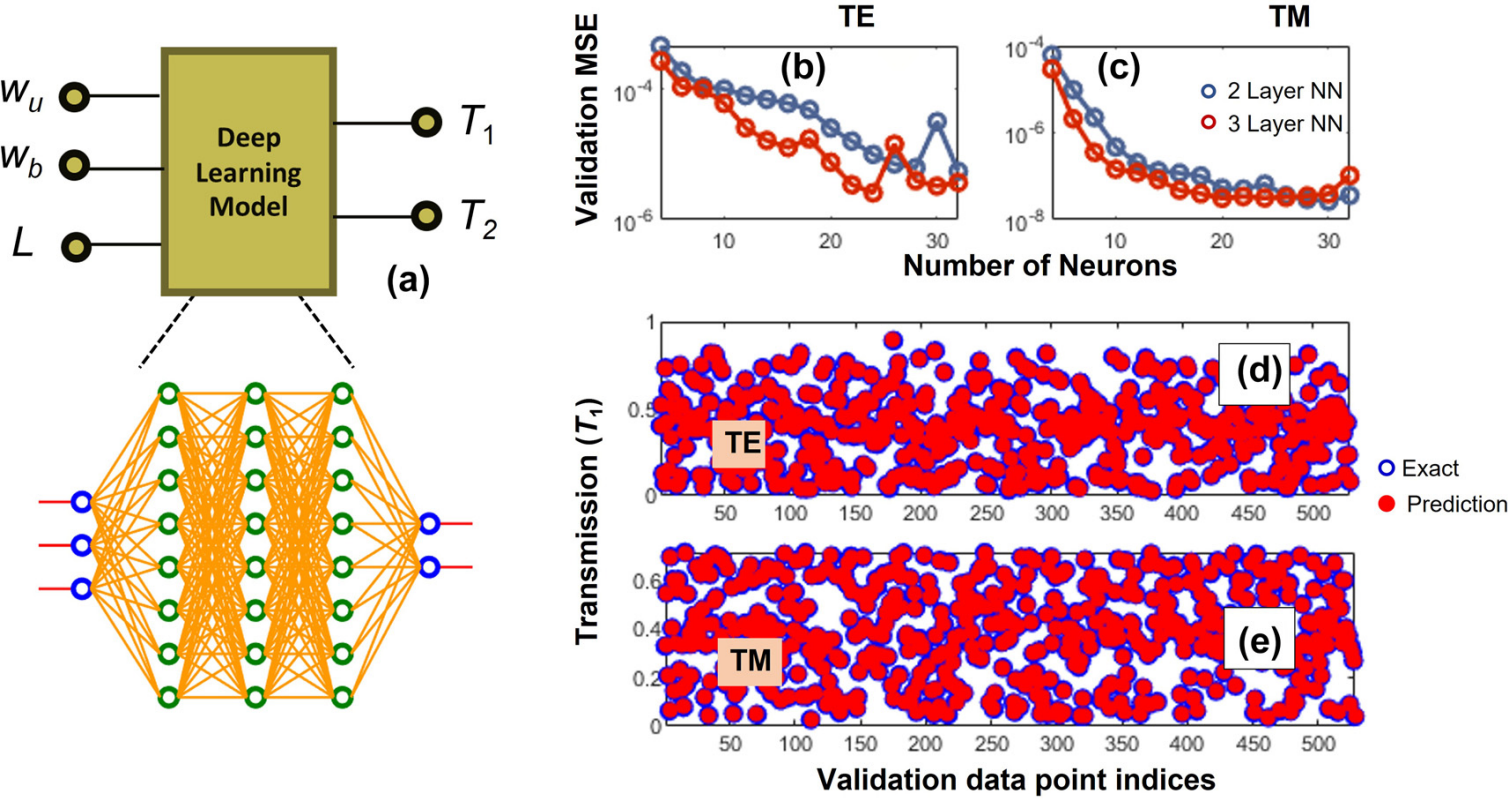
Deep learning based forward model. (a) The deep learning architecture with full feedforward connections. The validation mean-squared error (MSE) for (b) TE-polarization, and (c) TM-polarization. Transmission of the upper output waveguide for various geometries in the validation data set for (d) TE-polarization, and (e) TM-polarization. In (d) and (e), blue – exact values obtained from 3D FDTD; red – deep learning predictions.

Figure 3(a) showcases the deep learning model used in the forward modeling of the power divider. The inputs are the geometrical parameters [*w*_u_, *w*_b_, and *L*], and the outputs are the power transmissions of the two output waveguides [*T*_1_, *T*_2_]. The deep learning model contains densely connected layers with full feedforward connections [36] [see the bottom panel in Figure 3(a)]. The number of layers and the number of neurons in each layer are the hyperparameters. For training, a parameter space in which the geometrical parameters vary in the ranges of 0.25 µm < *w*_u_ < 1 µm, 0.25 µm < *w*_b_ < 1 µm and 1.6 µm < *L* < 3.6 µm is considered. We prepared a total of 2800 datapoints with transmission values calculated from the full three-dimensional (3D) FDTD methods [50, 51]. The datapoints are split into training and validation data sets with a split of ratio 0.8 and 0.2, respectively. The deep learning model is trained with Levenberg–Marquardt (LM) backpropagation algorithm, which is the fastest for a medium-size neural network [52, 53]. Figure 3(b) and (c) shows the mean square error in the validation data set as functions of hyperparameters for both TE and TM light polarizations. In this figure, the horizontal axis represents the number of neurons, and the red and blue lines, respectively, represent two- and three-layer neural networks. From these figures, we can see that for TE (TM) polarization, the optimized neural network architecture has validation error of 2.6 × 10^−6^ (3.1 × 10^−8^) for a threelayer network with 24 (20) neurons in each layer. Figure 3(d) and (e) shows the prediction (using the optimized neural network architecture) versus exact values of *T*_1_ for datapoints in the validation dataset for TE and TM polarizations, respectively. Figure 3(d) and (e) shows no visual discrepancy between the predictions and the exact values.

For the inverse modeling we explored pattern – search, particle swarm, simulated annealing, and genetic algorithms. These are well known nature-inspired optimization algorithms [38]. The objectives of fitting functions in these algorithms are set to minimize the quantity $\sqrt{{\left(R-T_{1}\right)}^{2}+{\left(1-R-T_{2}\right)}^{2}}$ at the design wavelength of 1550 nm. We also considered a multi-objective genetic algorithm with objectives of minimizing $\left|R-\frac{T_{1}}{T_{1}+T_{2}}\right|$ , $\left|1-R-\frac{T_{2}}{T_{1}+T_{2}}\right|$ , and $\left|1-T_{1}-T_{2}\right|$ . The objective functions are kept simple as to accelerate the entire inverse design process. The TE and TM polarizations mixing are checked in the final verification stage.

## 3 Programmable power splitting ratio

In this section, we present the inverse designs of power divider with an adjustable power splitting ratio. As shown, the inverse design reveals the relationship between the geometrical parameters and *R*. The relationship enables us to design power dividers with specific values of power splitting ratio.

**Figure 4:**
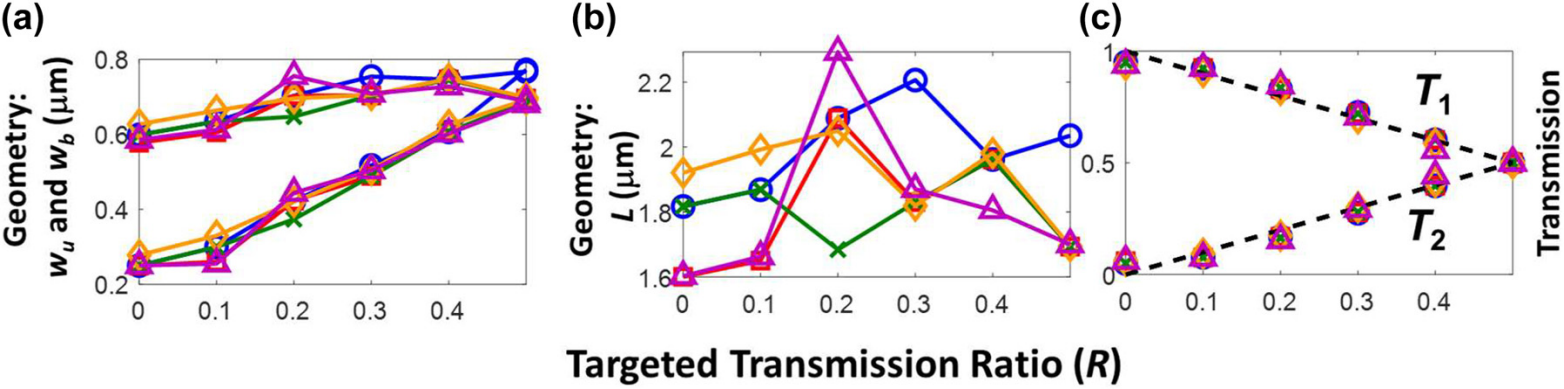
The geometries of the inverse-designed power divider with varying power splitting ratio. (a) Inverse-designed divider width profiles, *w*_u_ and *w*_b_ as a function of targeted transmission ratio (*R*). (b) Inverse-designed divider length, *L* as a function of targeted transmission ratio. (c) Transmissions *T*_1_ and *T*_2_ against the ideal values (solid lines). In (a)–(c), blue, red, orange, purple, and green represent pattern-search, particle swarm, simulated annealing, genetic, and multi-objective genetic algorithms, respectively.

In Figure 4, we show the results of execution of flow chart in Figure 2 for various *R*. Figure 4(a) and (b) plots the converged geometrical parameters, and Figure 4(c) shows their transmissions against the ideal values. We can see from Figures 4(a) and (b), there are slight variations in the final geometry [*w*_u_, *w*_b_: Figure 4(a); *L*: Figure 6(b)] discovered by the various algorithms. These variations are caused by the random nature of the algorithm which explores a wide parameter space spanned by the quantities *w*_u_, *w*_b_ and *L*. Different optimization algorithms, have varying stopping criteria such as maximum number of iterations, mesh tolerance, stalling of fitness functions etc., which in turn, is strenuous to control and force a final unified convergence in the wider parameter space. To overcome this problem, we confined the optimization to a smaller design space of fixed *L*, and repeated the optimization as a function of *L*.

From Figure 4(b), we can see that for all inverse designed devices the divider length varies between 1.6 and 2.2 µm. Now let us consider a divider with fixed length of *L* = 2 μm and demonstrate the programmability of the power divider. We can repeat this for another *L* for further optimizations on the loss, footprint, and bandwidth. This will be illustrated in next section.

Table 2 lists the inverse-designed widths *w*_u_, and *w*_b_ for *L* = 2 μm and *R* ranging from 0 to 0.5. The table tabulates these parameters for both TE and TM polarizations of light. As we can clearly see from the table all inverse algorithms converge to similar geometries. The fluctuations in the final geometrical parameters are smaller than the case of an *L* being a design parameter. The first three algorithms (pattern-search, particle swarm, and genetic) yields identical results for the final geometry (see Tables 1 and 2).

**Table 2:** The inverse-designed values of the geometrical parameters (*w*_u_, *w*_b_) for various *R* found by various inverse algorithms for a fixed *L* = 2 µm.

Inverse algorithm type	Inverse-designed *w*_b_ for *R* (TE pol)					
	0	0.1	0.2	0.3	0.4	0.5
Pattern search	0.2624	0.3138	0.4072	0.4831	0.6134	0.7629
Particle swarm	0.2624	0.3138	0.4072	0.4831	0.6134	0.7628
Genetic	0.2624	0.3138	0.4072	0.4831	0.6134	0.7629
Simulated annealing	0.2579	0.3166	0.4057	0.4836	0.6176	0.766
Multi-objective genetic	0.2593	0.335	0.4234	0.5156	0.624	0.7624

Inverse algorithm type	Inverse-designed *w*_u_ for *R* (TM pol)					
	0	0.1	0.2	0.3	0.4	0.5
Pattern search	0.6248	0.6548	0.6842	0.7122	0.7613	0.7628
Particle swarm	0.6248	0.6548	0.6842	0.7122	0.7613	0.7628
Genetic	0.6248	0.6548	0.6843	0.7122	0.7614	0.7628
Simulated annealing	0.6212	0.6666	0.6731	0.7473	0.7641	0.7631
Multi-objective genetic	0.6242	0.6581	0.6924	0.7179	0.7664	0.766

**Figure 5:**
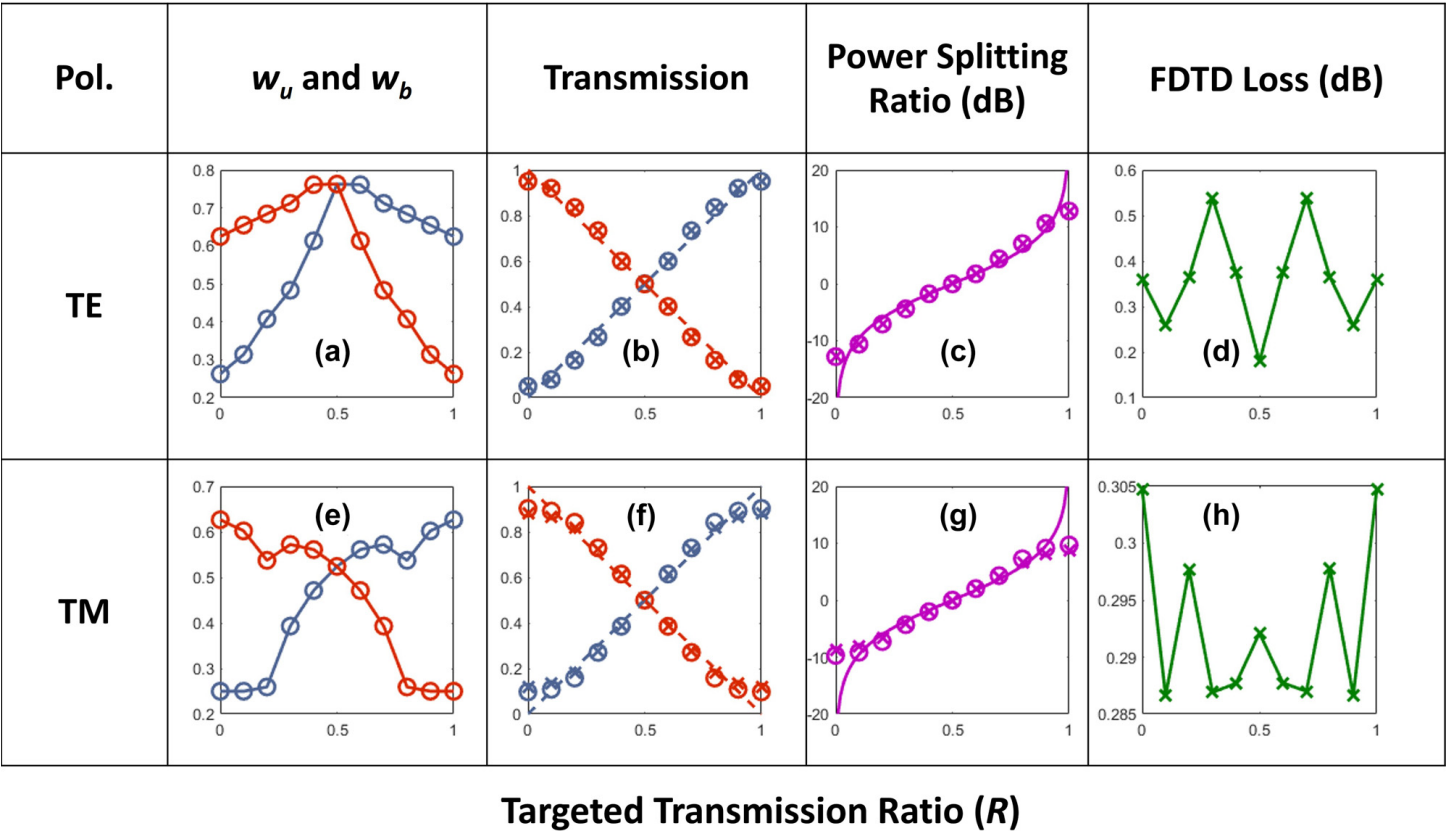
Graphical illustration of the inverse design results for *L* = 2 µm as a function of targeted transmission ratio (*R*). (a)–(d) TE-polarization. (a) Inverse-designed geometrical parameters. (b) The transmission for the devices with *w*_u_ and *w*_b_ as in (a). (c) The power splitting ratio for the devices with *w*_u_ and *w*_b_ as in (a). (d) The FDTD losses for the devices with *w*_u_ and *w*_b_ as in (a). In (b)–(d) lines: ideal values, circles: deep learning predictions, and crosses: 3D FDTD simulations. (e)–(h) Similar results as in (a)–(d) but for TM-polarization.

**Figure 6:**
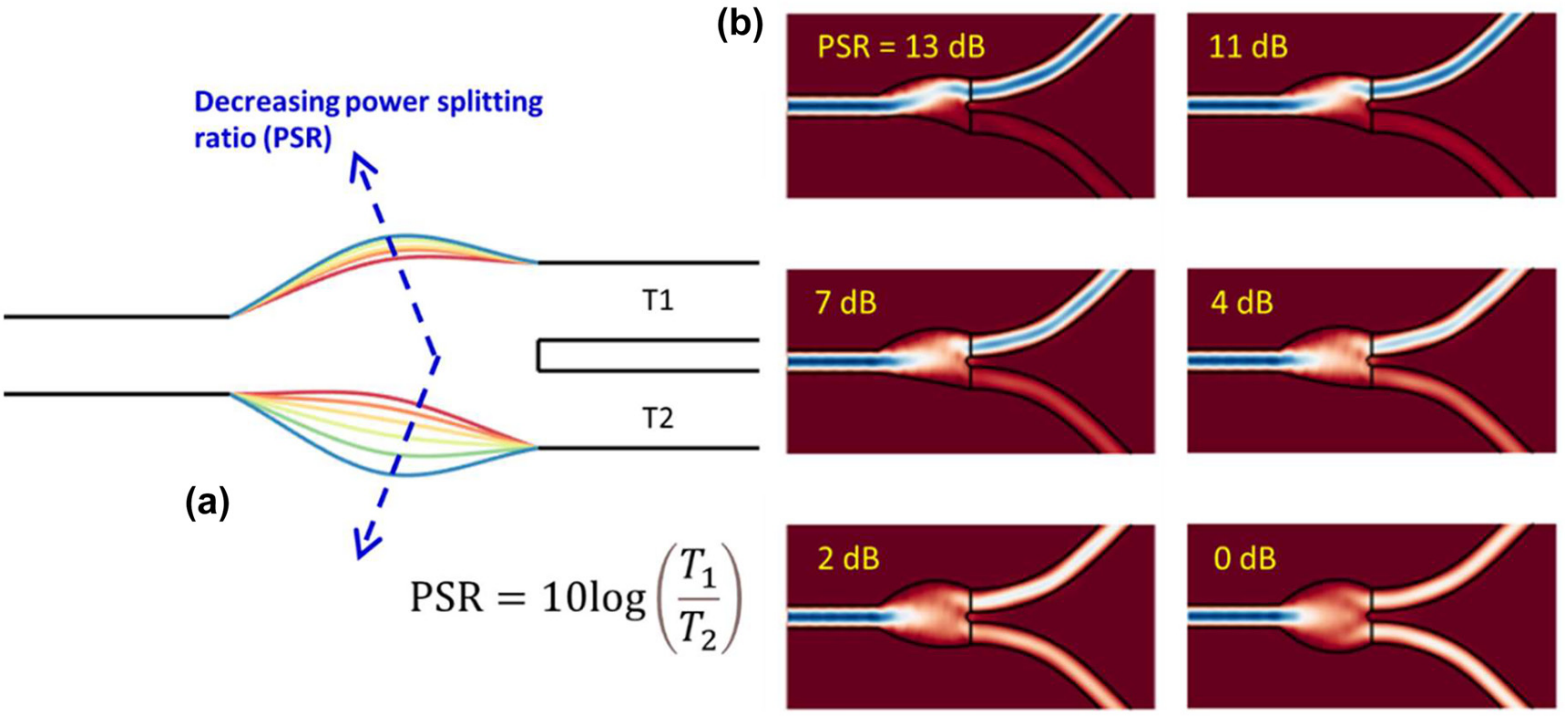
Inverse-designed silicon photonic power dividers. (a) Scaled view of the inverse-designed power dividers for various PSR levels. [Shown in different colors]. (b) FDTD simulations showing flow of power in the power dividers with various PSR levels.

Figure 5(a) and (e) graphically illustrates the converged values of *w*_u_, and *w*_b_ for these algorithms as functions of *R*. As can be seen from these figures, the widths versus *R* plots have clear mirror symmetries around *R* = 0.5 with $w_{\text{u}}\left(R\right)=w_{\text{b}}\left(1-R\right)$ . In Figure 6(a), we show the scaled planar view of the geometry of the inverse designed power divider as a function of power splitting ratio (PSR) for TE-polarization. Here, PSR is defined as $10\log\left(\frac{T_{1}}{T_{2}}\right)$ . From Figure 6(a), we can see that as PSR increases, the lower width profile of the junction bends upwards pushing more light to the upper output waveguide. Figure 6(b) illustrates the power flow across the power dividers with various PSR levels. The diagrams depict clear evidence of low loss power dividing operations in the inverse designed geometries. In Figure 5(b)–(d), we present physics-based numerical verifications (i.e., 3D FDTD) of the inverse designs for the transmissions and the power splitting ratio for the TE polarization of light. In Figure 5(b) and (c), the solid lines and open circles represent the ideal design values and deep learning predictions, respectively. The crosses on the other hand represent FDTD verified results. As can be seen from these figures deep learning predictions are in very good agreement with FDTD calculations. Figure 5(d), the FDTD evaluated excess loss is shown as a function of *R*. Figure 5(f)–(h) demonstrates similar results as Figure 5(b)–(d), however for TM polarization of light. For TE polarization, the FDTD losses of all devices with various *R* remains below 0.55 dB. On the other hand, for TM polarization the losses for all devices vary between 0.28 and 0.31 dB. In the rest of the article, for the sake of discussion we will only presents results for TE polarization. However, the methodology presented can be still applied for TM polarization.

**Figure 7:**
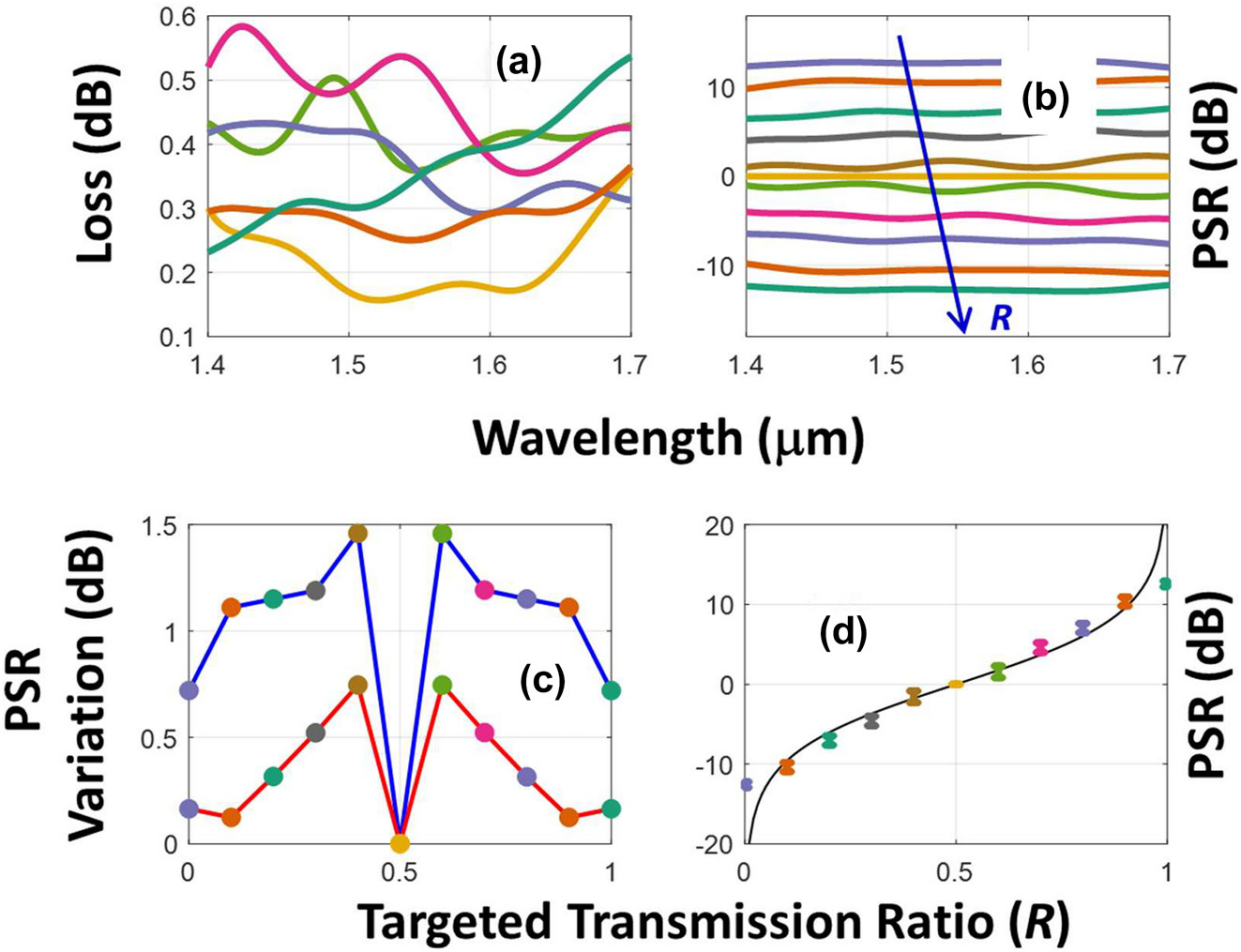
Wavelength response of the inverse-designed power dividers. (a) FDTD losses and (b) power splitting ratio as functions of wavelengths for various targeted transmission ratios. (c) Power splitting ratio variation across 300 nm (blue) and 100 (red) wavelength windows centered at 1550 nm. (d) Power splitting ratio variations across a 300 nm wavelength plotted on the ideal design curve. In all (a)–(d), similar color codes are used for devices with the same *R*. The color for each *R* can be identified using the respective marker colors from Figure 6(c) and (d).

Figure 7 depicts the wavelength response of the inverse-designed power dividers. All the wavelength responses were calculated using the 3D FDTD method. Figure 7(a) and (b) plots excess losses and PSR values as functions of wavelength for the devices of various *R*. We consider a wavelength window spanning a bandwidth of 300 nm and centered at 1.55 μm. Within this window, the losses of all devices are below 0.6 dB [Figure 7(a)]. On the other hand, the similar variation in PSR is 1.5 dB for all *R* [see the blue curve in Figure 7(c)]. For a narrower window of 100 nm (covering entire C band), the variation of PSR across the wavelengths is less than 0.6 dB for all *R* [see the red curve in Figure 7(c)]. This is an improvement of 0.4 dB over the state-of-the art where 1 dB variation of PSR (FDTD simulated) is reported for a similar 100 nm wavelength window [9]. Figure 7(d) depicts the wavelength variability of the PSR on the ideal design curves as a function *R*. Both losses and the corresponding wavelength span can be further optimized by repeating the analysis for another *L*, and this will be presented in the next section.

Before moving to the next section, let us briefly illustrate the computational effort taken by our deep learning assisted inverse algorithm. For computation we used MATLAB R2017a global optimization toolbox in Windows 10 workstation of Intel(R) Xeon(R) @ 2.33 MHz CPU [24 logical processors and 192 GB RAM]. Table 3 shows the computational time taken by various inverse algorithms for *R* = 0.5. In the second column of the table, we tabulated the quantity, *function count*, which denotes the number of times the inverse algorithm accesses the forward model. Columns 3 and 4 shows the respective inverse modelling time when the forward model being deep learning-based and FDTD-based, respectively. For FDTD, we assume each forward simulation consumes a 3-min calculation time. Clearly, the deep learning assisted inverse algorithms offers incomparable computational speed in secs as opposed to days in FDTD based models. The duration ranging to days reported in Table 3 is not surprising as similar computational time is reported earlier [8, 11, 40] for similar photonic device optimizations.

**Table 3:** Time required for inverse modelling. The third and fourth columns list the computational time when the forward model is being deep learning and FDTD-based models, respectively.

Inverse algorithm	Functioncount	Time – deep learning	Time – FDTD
Pattern search	430	4 s	∼0.9 days
Particle swarm	1440	13 s	∼3.0 days
Simulated annealing	2434	22 s	∼5.0 days
Genetic	3500	31 s	∼7.3 days

## 4 Loss, footprint, and bandwidth

**Figure 8:**
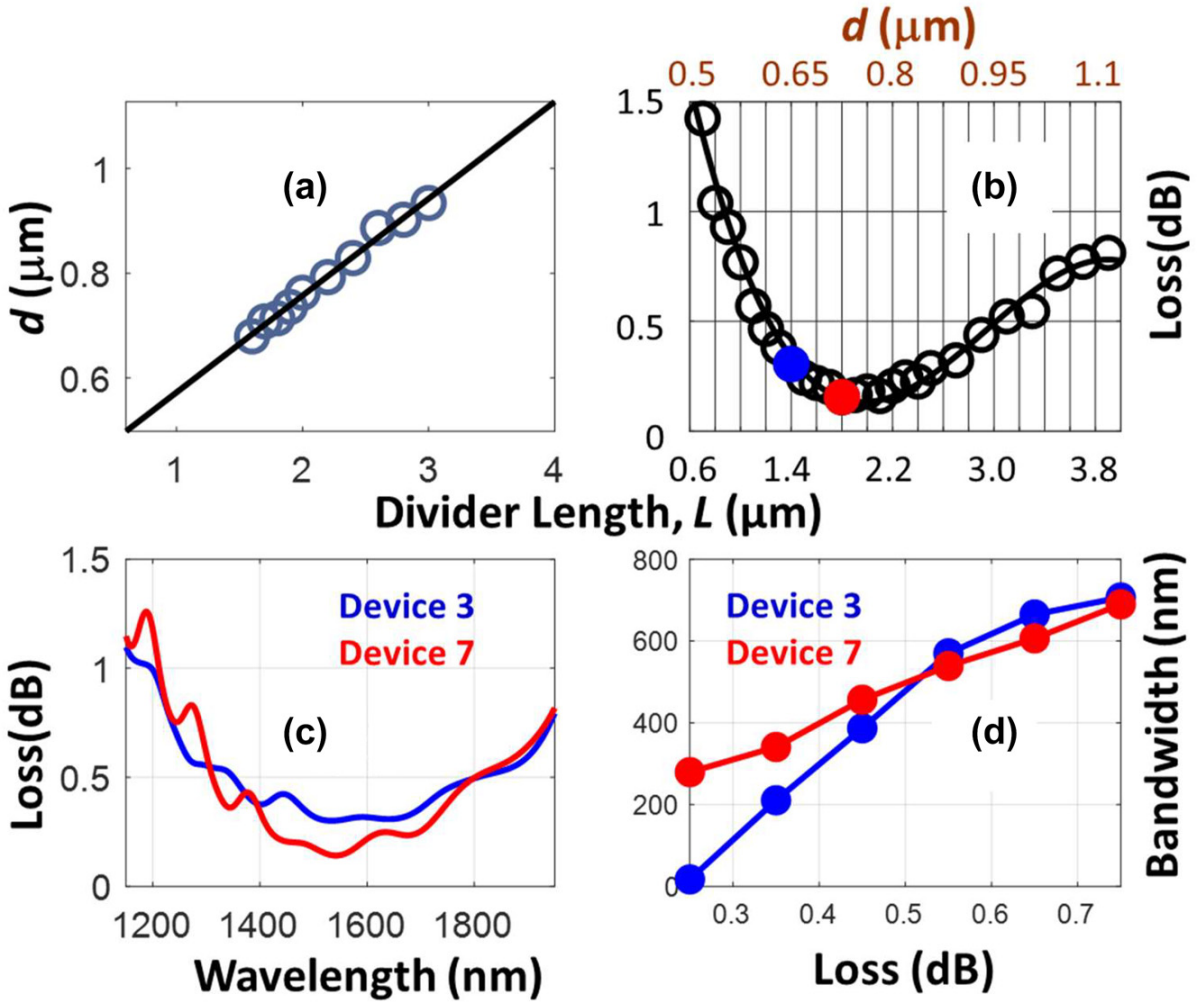
Performance of the equal power Y-junctions. (a) The geometries (*d* and *L*) of the inverse-designed Y-junctions (blue open circles). The solid linear line $\left\{d=0.185L+0.39\right\}$ is the fit to the blue circles. (b) The FDTD simulated loss as a function of geometrical parameters (circles). The solid line represents the fourth order polynomial fit for the circles. In this figure top and bottom horizontal axes represent *d* and *L*, respectively. The parameters *d* and *L* are linearly related [see (a)]. (c) The losses as functions of wavelength for devices with *L* = 1.4 µm (blue), and 1.8 µm (red). (d) Bandwidth as a function of loss levels for the two devices in (c).

**Figure 9:**
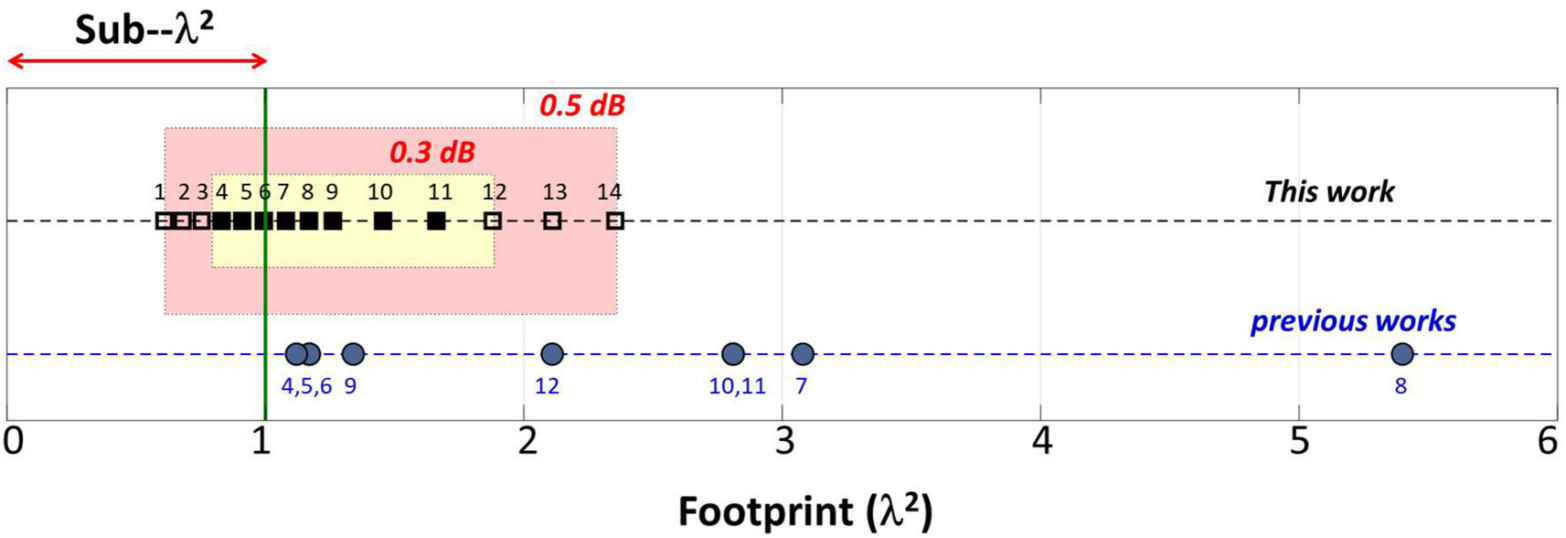
Spectrum of devices that simultaneously exhibit small footprints and low losses (orange region below 0.5 dB, yellow region: below 0.3 dB). The numbers on black dotted line represent the device numbers on Table 4. The numbers on the blue line are the reference numbers.

In this section, using an equal power Y-Junction $\left(R=0.5\right)$ as an example, we illustrate the simultaneous satisfaction of low loss, ultrawide bandwidth and sub-*λ* ^2^ footprint performance metrics. For an equal power Y-Junction, we have *w*_b_ = *w*_u_ = *d*. In Figure 8(a), we plotted the inverse designed values of *d* as a function of *L* using circles. The circles lie within the initial scope of *L*, 1.6 µm < *L* < 3.6 µm that is used to train the deep learning-based forward model. As we can see from Figure 8(a), the circles can be fitted with a linear line of equation *d* = 0.185*L* + 0.39. In Figure 8(a), we show this line for a wider range, 0.5 μm < *L* < 4 μm. Figure 8(b) shows the FDTD simulated excess loss for a series of devices within this new range of *L*, with *d* obtained from the linear fit. In this figure, the solid line represents fourth-order polynomial fit for the FDTD simulated loss. Within the space described by the linear line *d* = 0.185*L* + 0.39, sub 0.5 dB losses occur for devices in the subspace: 1.2 μm < *L* < 3 μm [see Figure 8(b)]. We define footprint as a rectangular area, *A* = 2 *dL* = 0.37 *L* ^2^ + 0.78*L* that encapsulate the power divider. Therefore, the footprint of the devices with sub-0.5 dB losses fall in the range, 0.61*λ* ^2^ < *A* < 2.36*λ* ^2^ with *λ* = 1.55 µm. Table 4 lists the performance of the representative devices with losses below 0.5 dB. In the fourth column of this table, we tabulate the footprint in the units of *λ* ^2^. Device number 7 exhibit the lowest excess loss of 0.14 dB with a footprint of ∼*λ* ^2^. We also note from Table 2 that there is a spectrum of devices that simultaneously exhibit sub-*λ* ^2^ footprints with excess losses below 0.5 dB. This occurs for 1.2 < *L* < 1.8 μm. In Figure 9, we graphically display the region of *A* in which devices with sub 0.5 dB and sub-0.3 dB devices occur. The square markers in the device are the footprints of the representative devices from Table 4. The sub-*λ* ^2^ devices shown here are the smallest devices with ultralow FDTD losses reported so far. In Figure 9, we have also compared the footprints of the earlier state of arts (see Table 1) with sub 0.5 dB FDTD losses.

**Table 4:** The performances of the representative devices with sub-0.5 dB losses a function of geometrical parameters and footprints.

#	*L* (µm)	*d* (µm)	*A* =2 *d*L (λ^2^)	Loss (dB) at 1.55 µm	Bandwidth (nm) for loss levels of			
					0.25 dB	0.35 dB	0.55 dB	0.75 dB
**1**	1.2	0.61	**0.61**	**0.47**	0	0	456	680
**2**	1.3	0.63	**0.68**	**0.39**	0	0	561	701
**3**	1.4	0.65	**0.75**	**0.30**	0	212	569	706
**4**	1.5	0.67	**0.83**	**0.24**	46	337	554	678
**5**	1.6	0.68	**0.91**	**0.21**	181	373	548	694
**6**	1.7	0.70	**0.99**	**0.19**	210	338	598	673
**7**	1.8	0.72	**1.08**	**0.14**	252	341	538	678
**8**	1.9	0.74	**1.17**	**0.15**	196	396	491	644
**9**	2	0.76	**1.26**	**0.17**	248	301	526	601
**10**	2.2	0.79	**1.46**	**0.19**	172	265	413	555
**11**	2.4	0.83	**1.66**	**0.23**	109	213	354	485
**12**	2.6	0.87	**1.88**	**0.35**	0	133	287	420
**13**	2.8	0.91	**2.11**	**0.40**	0	0	234	356
**14**	3	0.94	**2.35**	**0.46**	0	0	178	303

In the columns 6–9 of Table 4, we have tabulated the bandwidth of the devices for loss levels of 0.25 dB, 0.35 dB, 0.55 dB, and 0.75 dB. The largest bandwidth of 252 nm with lowest loss (0.25 dB) is obtained for device number 7 (highlighted in red). The bandwidth of the devices increases when the specified loss level is higher. Device number 3 (highlighted in blue), with *L* = 1.4 μm and the sub-*λ* ^2^ footprint *A* = 0.75*λ* ^2^, exhibits the largest bandwidth of ∼700nm with the loss of 0.75 dB. For loss level of 0.55 dB, this device exhibits a bandwidth of 569 nm. Figure 8(d) graphically illustrates the evolution of the bandwidth as a function of loss level for devices $3\left(L=1.4\;\mu \text{m}\right)$ and $7\left(L=1.8\;\mu \text{m}\right)$ . Their wavelength spectrums are shown in Figure 8(c).

The telecommunication wavelength window can be subdivided into ordinary(O): 1260–1360 nm, extended(E): 1360–1460 nm, short (S): 1460–1530 nm, conventional(C): 1530–1565 nm, long (L): 1565–1625 nm, and ultralong(U): 1625–1675 nm wavelength bands. In Table 5, we tabulate the maximum losses across all these bands for the devices presented in Table 4. As we can clearly see for all the tabulated devices, the maximum losses stay at or below 0.5 dB for the whole C-band. Devices with numbers 7 to 9 exhibit sub 0.2 dB loss across the entire C-band. These devices also display losses below 0.5 dB for all telecommunication bands excluding O-band. Device number 3 (highlighted in blue) has the lowest loss covering the entire band from O to U. This device exhibits losses of 0.61 dB, 0.31 dB and 0.32 dB for O-, C-, and U-band, respectively.


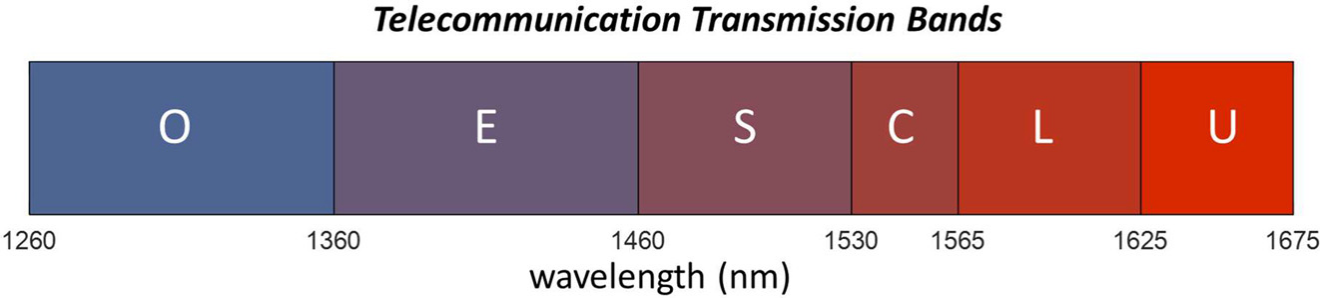


**Table 5:** The maximum losses of devices in Table 2 for various telecommunication bands.

#	*L* (µm)	*d* (µm)	*A* =2 *d*L (λ^2^)	Loss (dB) at 1.55 µm	Loss (dB)					
					O	E	S	C	L	U
**1**	1.2	0.61	**0.61**	0.47	0.66	0.56	0.54	**0.49**	0.49	0.52
**2**	1.3	0.63	**0.68**	0.39	0.68	0.53	0.43	**0.40**	0.38	0.43
3	1.4	0.65	**0.75**	0.30	0.61	0.47	0.41	0.31	0.32	0.32
**4**	1.5	0.67	**0.83**	0.24	0.76	0.49	0.30	**0.25**	0.29	0.31
**5**	1.6	0.68	**0.91**	0.21	0.68	0.40	0.24	**0.23**	0.23	0.32
**6**	1.7	0.70	**0.99**	0.19	0.75	0.44	0.26	**0.20**	0.21	0.25
**7**	1.8	0.72	**1.08**	0.14	0.83	0.43	0.20	**0.16**	0.25	0.25
**8**	1.9	0.74	**1.17**	0.15	0.73	0.34	0.19	**0.16**	0.23	0.33
**9**	2.0	0.76	**1.26**	0.17	0.95	0.49	0.22	**0.18**	0.19	0.32
**10**	2.2	0.79	**1.46**	0.19	1.05	0.46	0.21	**0.21**	0.29	0.39
**11**	2.4	0.83	**1.66**	0.23	1.11	0.45	0.24	**0.26**	0.36	0.43
**12**	2.6	0.87	**1.88**	0.35	1.09	0.57	0.31	**0.38**	0.43	0.61
**13**	2.8	0.91	**2.11**	0.40	1.13	0.65	0.40	**0.40**	0.62	0.66
**14**	3.0	0.94	**2.35**	0.46	1.20	0.70	0.45	**0.50**	0.68	0.86

### 4.1 Comparison with earlier works

In Table 1, we have summarized the performances of various earlier reported photonic Y-junctions on the standard single-mode silicon-on-insulator platform with a silicon thickness of 220 nm. The table is compiled using the reported simulation values from Refs. [5–13]. Devices with sub 0.5 dB losses are reported in Refs. [4], [5], [6, 9], [10], [11], [12]. The footprints of all these devices are graphically shown in Figure 9. None of these devices have footprint below *λ* ^2^. While in our case, we have found a region in the design space [*L*: 1.2 < *L* < 1.8 μm, *w*_b_ = *w*_u_ = *d*: *d* = 0.185*L* + 0.39] that simultaneously satisfy both sub-*λ* ^2^ and sub-0.5 dB loss conditions. Devices 1–6 are representative devices in this region and their performances are tabulated in Tables 4 and 5, and graphically illustrated in Figure 9. Device 1 has the smallest footprint of 0.61*λ* ^2^. In addition to the loss and footprint, our approach produces devices with wider bandwidth. The bandwidths and the losses of the devices 2 to 7 are comparable to the state-of-art device with the largest bandwidth of 550 nm [12] at ∼0.5 dB loss (see Table 1). This can be seen from by examining the bandwidths at the closer loss level of ∼0.55 dB in Table 4. However, in terms of the footprints of the devices 2 to 7 are 48%–67% smaller than the device in Ref. [12] which has a footprint of 2.11*λ* ^2^.

Another important aspect of design performance that was less discussed in earlier literatures is robustness. Our devices exhibit robust performances. For a tolerance level as large as 50 nm in the critical parameter, the standard deviation of the loss distribution of our devices is half of the standard deviation of the earlier state-of-art [5]. The details of pertaining statistical study will be given in the next section. The robustness is the direct consequence of our simplistic base geometry for the proposed power divider. The proposed power divider has smooth width profiles constructed using cubic spline interpolation. This is the most simplistic geometry in comparison to all previous demonstrations. As explained earlier in the introduction, topology of earlier reported structures possesses complicated features such as fine variation in the width profile [5, 6] and optimized 2D hole vectors [7, 8, 10], [11], [12].

Throughout this article, for the purpose of a fair discussion, we have compared key works of power dividers in the form Y-junctions on the standard silicon-on-insulator platform with a silicon thickness of 220 nm. The input and output waveguides are single modal and has widths of 500 nm. There is also other non-routine inverse designed power divider works reported in the literature. For examples, in Ref. [14], a sparse parameter method is used to build power divider for 400 nm silicon waveguide with air cladding. Refs. [15, 16] report silicon power dividers with 1 × 3 power splitters. There is also inverse design works of power dividers in the form of T-junctions [13], and multimodal power splitters which can split more than one waveguide mode [17, 18].

## 5 Robustness against fabrication randomness

Now let us demonstrate the robustness of the discovered power dividers with respect to the random variations in their geometrical design values. For the sake of illustration, let us consider the inverse-designed equal power Y-junction and compare it with the earlier work [5]. The device in Ref. [5] possesses a divider of length *L* = 2 µm and exhibits a low numerical loss around ∼0.1 dB at the wavelength of 1.55 µm. We compare this device against our inverse design for *L* = 2 µm [Device 9 in Tables 4 and 5]. For the convenience of referencing, let us call the earlier reported device and the current inverse design as *A*_ref_ and *A*_id_, espectively.

**Figure 10:**
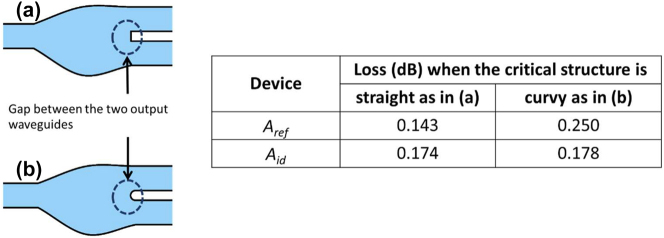
The structure in the proximity of the gap between the two output waveguides (a) straight, (b) curvy. The FDTD losses for the devices *A*_id_ and *A*_ref_ when the structure in vicinity of the gap is assumed as (a) and (b).

We will consider two robustness analysis. The first is on the fabrication of the gap between the two output waveguides [see Figure 10]. In the design, the structure in the vicinity of the gap is in rectangular form [Figure 10(a)], which is difficult to fabricate and due to stitching and rounding, the resultant fabricated structure would typically appear curvy as illustrated in Figure 10(b). Assuming the curvy structure is a semicircle of radius 0.5*D*, where *D* = 200 nm (see Figure 1) is the separation between the two output waveguides, we re-simulated *A*_ref_ and *A*_id_, and the FDTD simulated losses are shown in the insert of Figure 10. As we can clearly see the changes in loss for *A*_id_ is about 0.004 dB (2%), whereas the change in the loss for *A*_ref_ is about 0.1 dB (71%). This gives a clear indication of robustness of *A*_id_ in comparison *A*_ref_ with respect to a small structural modification.

**Figure 11:**
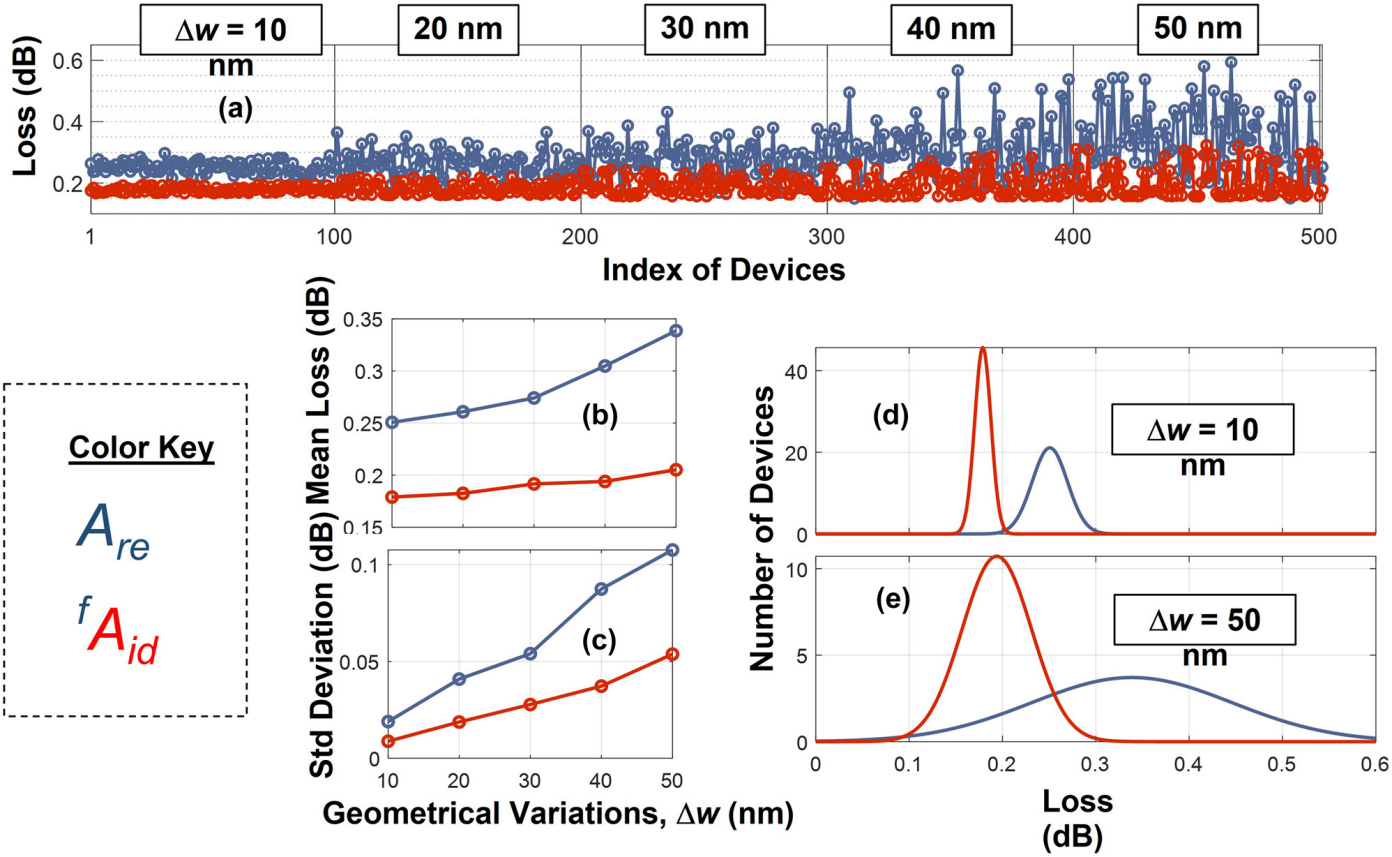
Robustness of devices *A*_ref_ (blue) and *A*_id_ (red). (a) The FDTD losses of 500 devices with identical design but with random fluctuations of Δ*w* in their critical parameters. (b) Mean losses and (c) standard deviation of losses as functions of Δ*w*. The fitted normal probability distribution functions for (d) Δ*w* = 10 nm and (e) Δ*w* = 50 nm.

The second robustness investigation were done by introducing randomness in the critical design parameters while keeping the curvy structures as in Figure 10(b). For *A*_id_, the critical parameter is the width of the waveguide taper, *w*_b_ = *w*_u_ = *d*. On the other hand, for *A*_ref_ there are 13 critical parameters defining the waveguide taper, and as we will show the large number of parameters in *A*_ref_ leads to lack of robustness against large-scale fabrications. We conducted the numerical experiment as follows: A total of 500 devices of *A*_id_ and *A*_ref_ with identical designs were considered. Random fluctuations were introduced in the ideal values of the critical parameters to assess the robustness. The FDTD losses of all 500 devices are shown in Figure 11(a). Each hundred devices in the figure have random deviations up to Δ*w* nm. For an example, the first hundred devices in Figure 11(a) have random variations up to 10 nm in their critical parameters. The similar variation for the next hundred devices is 20 nm, and so on. The mean and standard deviations as functions of Δ*w* are shown in Figure 11(b) and (c), respectively. From Figure 11(c), we can see that for a tolerance level as large as 50 nm in the critical parameter, the standard deviation of the loss distribution of our devices is roughly half of the standard deviation of the earlier state-of-art. Figure 11(d) and (e) shows the fitted normal probability distributions functions for the cases of Δ*w* = 10 nm and 50 nm. Figure 11(a)–(e) clearly demonstrates the robustness for our devices in comparison to the current state-of-art design. As we can clearly see, our design $\left[A_{\text{id}}\right]$ remains robust with maximum loss as small as 0.3 dB [∼0.13 dB deviation from the ideal value 0.174 dB (Figure 10)] in spite of a large random variation of Δ*w* = 50 nm. The corresponding maximum loss for *A*_ref_ is 0.6 dB [∼0.46 dB deviation from the ideal value 0.143 dB (Figure 10)]. This indicates our design is able to reduce maximum loss by over 50% despite a large 50 nm random fabrication variation. In turn, these findings can help sustainable large-scale fabrication that in highly tolerant to large fabrication process fluctuations.

## 6 Conclusions

We present precise deep learning models for silicon photonic power dividers with tailorable power splitting ratio. The width profile of the divider is defined by a cubic spline interpolant of two parameters. The deep learning models have validation mean squared errors on the order of 10^−6^ to 10^−8^ for both TE and TM polarizations of light. Using the deep learning model as the forward model, we conducted an ultrafast search for subspace of performance excellence using inverse design algorithms. The general algorithm and specific results for the design of power dividers with adjustable power ratios are presented. Using, an equal power Y-junction as an illustrative example, we show a discovery of subspace of devices that simultaneously satisfy various performance metrics. The discovered devices simultaneously satisfy compact footprints (on the order of sub-λ^2^), ultralow insertion losses (below 0.5 dB), ultrawide bandwidth (more than 500 nm; covering a wide range of telecommunication bands) and exceptional robustness against fabrication randomness. The performance metrics of the deep learning-based designs are in perfect agreement with the full three-dimensional finite difference time domain calculation.
